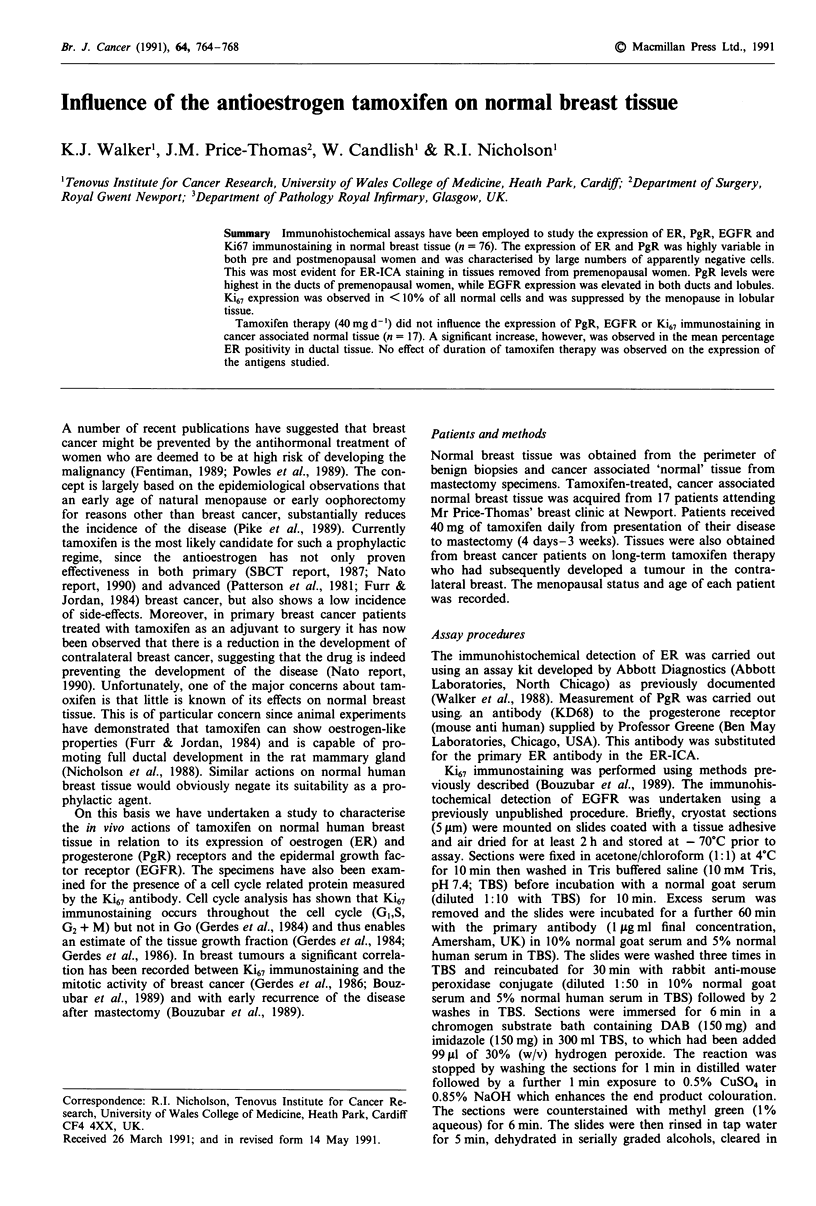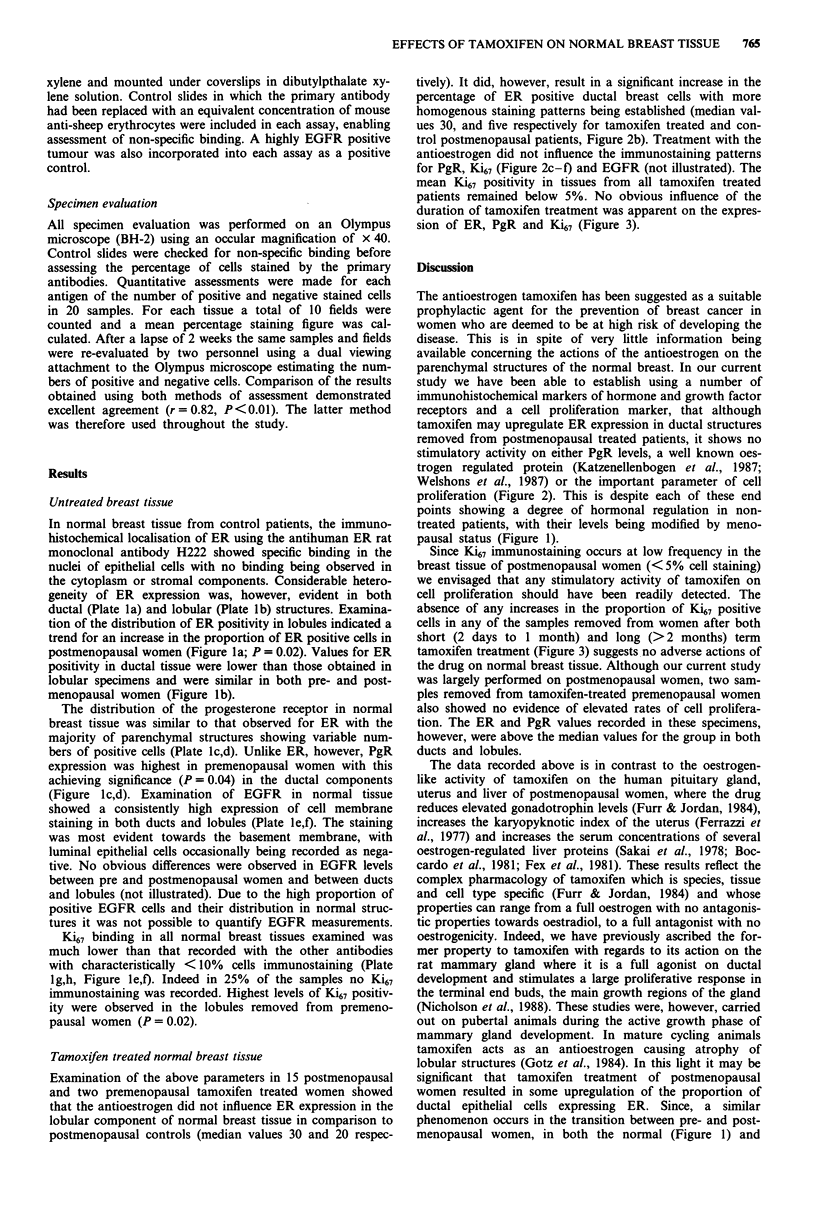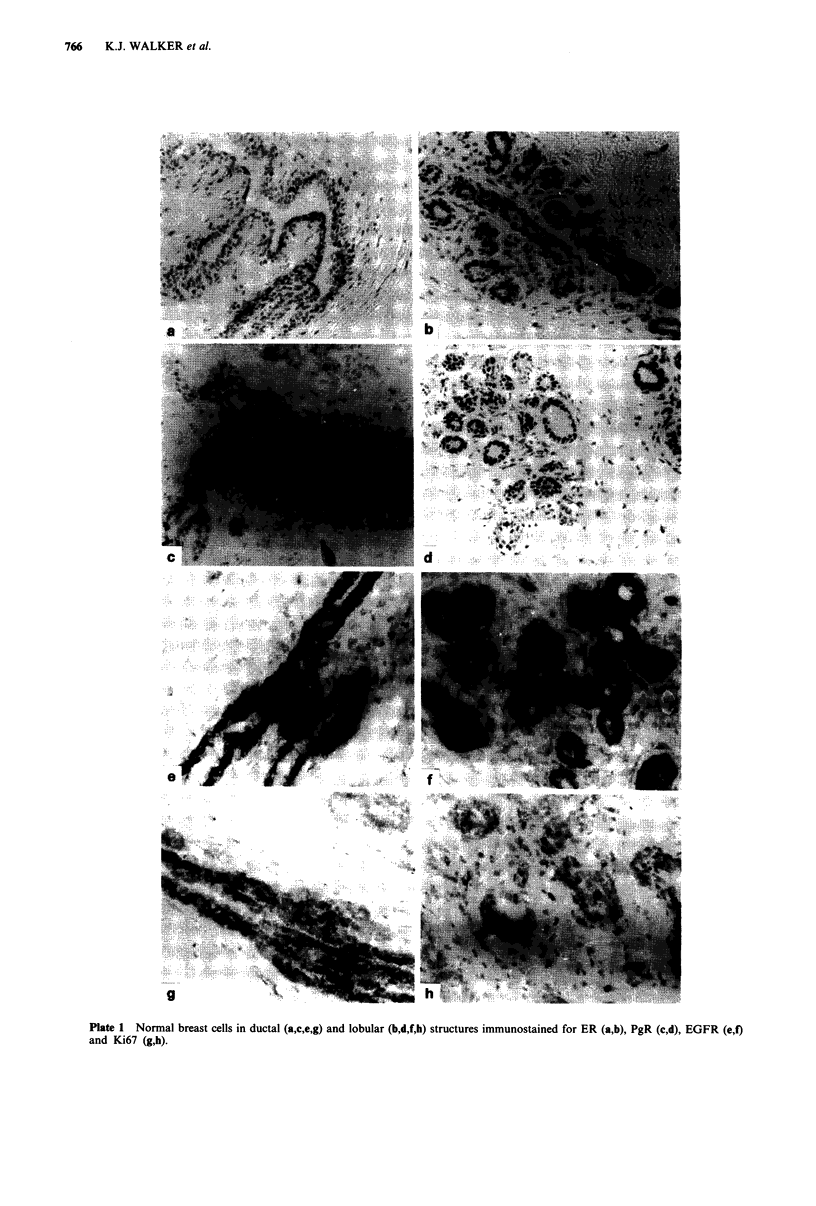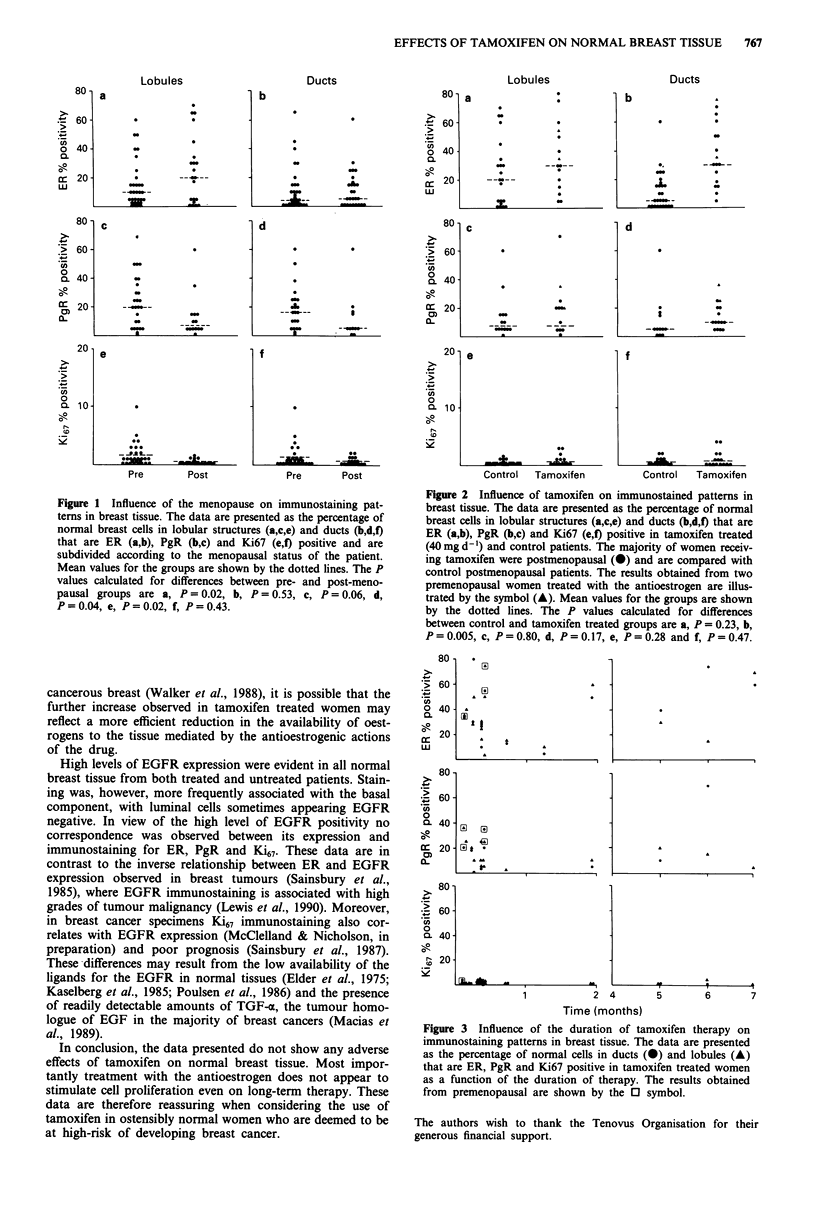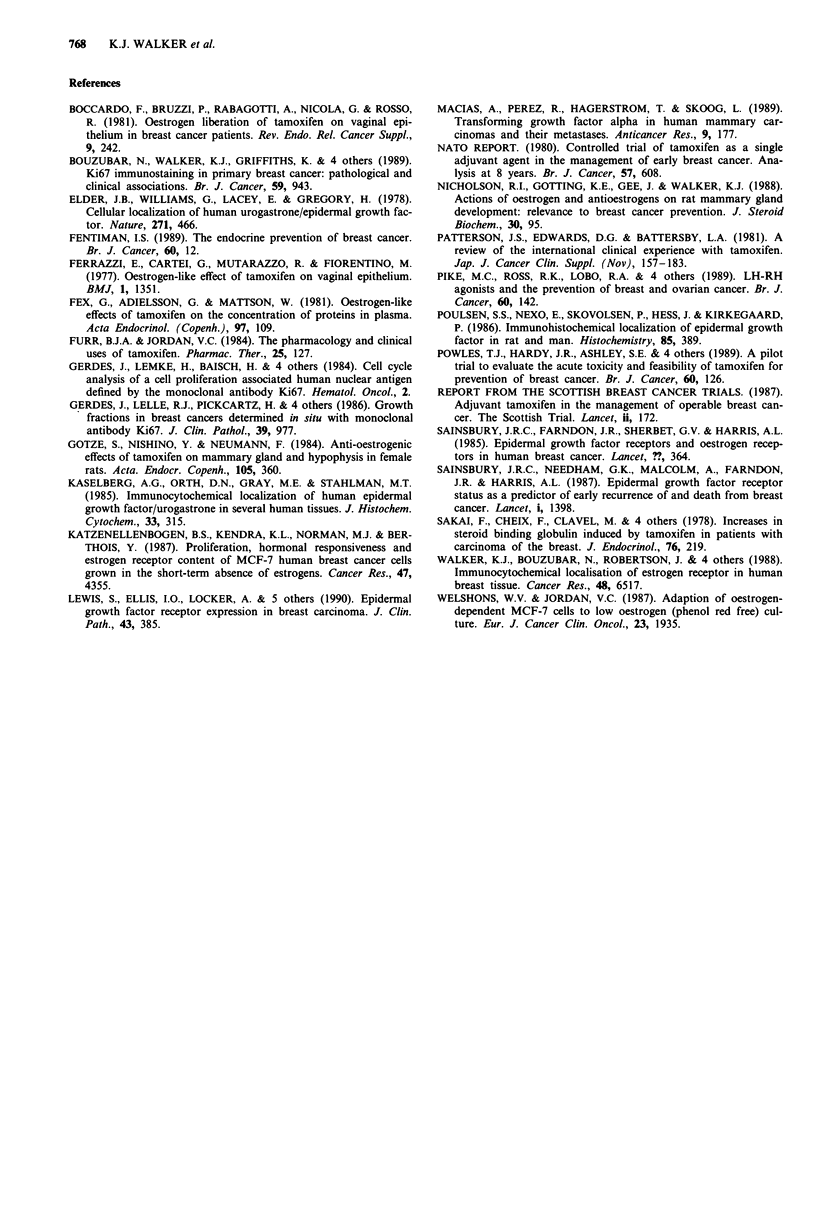# Influence of the antioestrogen tamoxifen on normal breast tissue.

**DOI:** 10.1038/bjc.1991.395

**Published:** 1991-10

**Authors:** K. J. Walker, J. M. Price-Thomas, W. Candlish, R. I. Nicholson

**Affiliations:** Tenovus Institute for Cancer Research, University of Wales College of Medicine, Heath Park, Cardiff, UK.

## Abstract

**Images:**


Br. J. Cancer (1991), 64, 764-768 © Macmillan Press Ltd., 1991

### Influence of the antioestrogen tamoxifen on normal breast tissue

K.J. Walker[1], J.M. Price-Thomas[2], W. Candlish[1] & R.I. Nicholson[1]

[1]*Tenovus Institute for Cancer Research, University of Wales College of Medicine, Heath Park, Cardiff;* [2]*Department of Surgery, Royal Gwent Newport;* [3]*Department of Pathology Royal Infirmary, Glasgow, UK.*

**Summary** Immunohistochemical assays have been employed to study the expression of ER, PgR, EGFR and Ki67 immunostaining in normal breast tissue ($n = 76$). The expression of ER and PgR was highly variable in both pre and postmenopausal women and was characterised by large numbers of apparently negative cells. This was most evident for ER-ICA staining in tissues removed from premenopausal women. PgR levels were highest in the ducts of premenopausal women, while EGFR expression was elevated in both ducts and lobules. $Ki_{67}$ expression was observed in $<10\%$ of all normal cells and was suppressed by the menopause in lobular tissue.

Tamoxifen therapy ($40\ mg\ d^{-1}$) did not influence the expression of PgR, EGFR or $Ki_{67}$ immunostaining in cancer associated normal tissue ($n = 17$). A significant increase, however, was observed in the mean percentage ER positivity in ductal tissue. No effect of duration of tamoxifen therapy was observed on the expression of the antigens studied.

A number of recent publications have suggested that breast cancer might be prevented by the antihormonal treatment of women who are deemed to be at high risk of developing the malignancy (Fentiman, 1989; Powles *et al.*, 1989). The concept is largely based on the epidemiological observations that an early age of natural menopause or early oophorectomy for reasons other than breast cancer, substantially reduces the incidence of the disease (Pike *et al.*, 1989). Currently tamoxifen is the most likely candidate for such a prophylactic regime, since the antioestrogen has not only proven effectiveness in both primary (SBCT report, 1987; Nato report, 1990) and advanced (Patterson *et al.*, 1981; Furr & Jordan, 1984) breast cancer, but also shows a low incidence of side-effects. Moreover, in primary breast cancer patients treated with tamoxifen as an adjuvant to surgery it has now been observed that there is a reduction in the development of contralateral breast cancer, suggesting that the drug is indeed preventing the development of the disease (Nato report, 1990). Unfortunately, one of the major concerns about tamoxifen is that little is known of its effects on normal breast tissue. This is of particular concern since animal experiments have demonstrated that tamoxifen can show oestrogen-like properties (Furr & Jordan, 1984) and is capable of promoting full ductal development in the rat mammary gland (Nicholson *et al.*, 1988). Similar actions on normal human breast tissue would obviously negate its suitability as a prophylactic agent.

On this basis we have undertaken a study to characterise the *in vivo* actions of tamoxifen on normal human breast tissue in relation to its expression of oestrogen (ER) and progesterone (PgR) receptors and the epidermal growth factor receptor (EGFR). The specimens have also been examined for the presence of a cell cycle related protein measured by the $Ki_{67}$ antibody. Cell cycle analysis has shown that $Ki_{67}$ immunostaining occurs throughout the cell cycle ($G_1$,S, $G_2$ + M) but not in Go (Gerdes *et al.*, 1984) and thus enables an estimate of the tissue growth fraction (Gerdes *et al.*, 1984; Gerdes *et al.*, 1986). In breast tumours a significant correlation has been recorded between $Ki_{67}$ immunostaining and the mitotic activity of breast cancer (Gerdes *et al.*, 1986; Bouzubar *et al.*, 1989) and with early recurrence of the disease after mastectomy (Bouzubar *et al.*, 1989).

Correspondence: R.I. Nicholson, Tenovus Institute for Cancer Research, University of Wales College of Medicine, Heath Park, Cardiff CF4 4XX, UK.
Received 26 March 1991; and in revised form 14 May 1991.

### *Patients and methods*

Normal breast tissue was obtained from the perimeter of benign biopsies and cancer associated 'normal' tissue from mastectomy specimens. Tamoxifen-treated, cancer associated normal breast tissue was acquired from 17 patients attending Mr Price-Thomas' breast clinic at Newport. Patients received 40 mg of tamoxifen daily from presentation of their disease to mastectomy (4 days–3 weeks). Tissues were also obtained from breast cancer patients on long-term tamoxifen therapy who had subsequently developed a tumour in the contralateral breast. The menopausal status and age of each patient was recorded.

### *Assay procedures*

The immunohistochemical detection of ER was carried out using an assay kit developed by Abbott Diagnostics (Abbott Laboratories, North Chicago) as previously documented (Walker *et al.*, 1988). Measurement of PgR was carried out using an antibody (KD68) to the progesterone receptor (mouse anti human) supplied by Professor Greene (Ben May Laboratories, Chicago, USA). This antibody was substituted for the primary ER antibody in the ER-ICA.

$Ki_{67}$ immunostaining was performed using methods previously described (Bouzubar *et al.*, 1989). The immunohistochemical detection of EGFR was undertaken using a previously unpublished procedure. Briefly, cryostat sections (5 µm) were mounted on slides coated with a tissue adhesive and air dried for at least 2 h and stored at − 70°C prior to assay. Sections were fixed in acetone/chloroform (1:1) at 4°C for 10 min then washed in Tris buffered saline (10 mM Tris, pH 7.4; TBS) before incubation with a normal goat serum (diluted 1:10 with TBS) for 10 min. Excess serum was removed and the slides were incubated for a further 60 min with the primary antibody (1 µg ml final concentration, Amersham, UK) in 10% normal goat serum and 5% normal human serum in TBS). The slides were washed three times in TBS and reincubated for 30 min with rabbit anti-mouse peroxidase conjugate (diluted 1:50 in 10% normal goat serum and 5% normal human serum in TBS) followed by 2 washes in TBS. Sections were immersed for 6 min in a chromogen substrate bath containing DAB (150 mg) and imidazole (150 mg) in 300 ml TBS, to which had been added 99 µl of 30% (w/v) hydrogen peroxide. The reaction was stopped by washing the sections for 1 min in distilled water followed by a further 1 min exposure to 0.5% $CuSO_4$ in 0.85% NaOH which enhances the end product colouration. The sections were counterstained with methyl green (1% aqueous) for 6 min. The slides were then rinsed in tap water for 5 min, dehydrated in serially graded alcohols, cleared in xylene and mounted under coverslips in dibutylpthalate xylene solution. Control slides in which the primary antibody had been replaced with an equivalent concentration of mouse anti-sheep erythrocytes were included in each assay, enabling assessment of non-specific binding. A highly EGFR positive tumour was also incorporated into each assay as a positive control.

### *Specimen evaluation*

All specimen evaluation was performed on an Olympus microscope (BH-2) using an occular magnification of $\times 40$. Control slides were checked for non-specific binding before assessing the percentage of cells stained by the primary antibodies. Quantitative assessments were made for each antigen of the number of positive and negative stained cells in 20 samples. For each tissue a total of 10 fields were counted and a mean percentage staining figure was calculated. After a lapse of 2 weeks the same samples and fields were re-evaluated by two personnel using a dual viewing attachment to the Olympus microscope estimating the numbers of positive and negative cells. Comparison of the results obtained using both methods of assessment demonstrated excellent agreement ($r = 0.82$, $P < 0.01$). The latter method was therefore used throughout the study.

### Results

### *Untreated breast tissue*

In normal breast tissue from control patients, the immunohistochemical localisation of ER using the antihuman ER rat monoclonal antibody H222 showed specific binding in the nuclei of epithelial cells with no binding being observed in the cytoplasm or stromal components. Considerable heterogeneity of ER expression was, however, evident in both ductal (Plate 1a) and lobular (Plate 1b) structures. Examination of the distribution of ER positivity in lobules indicated a trend for an increase in the proportion of ER positive cells in postmenopausal women (Figure 1a; $P = 0.02$). Values for ER positivity in ductal tissue were lower than those obtained in lobular specimens and were similar in both pre- and postmenopausal women (Figure 1b).

The distribution of the progesterone receptor in normal breast tissue was similar to that observed for ER with the majority of parenchymal structures showing variable numbers of positive cells (Plate 1c,d). Unlike ER, however, PgR expression was highest in premenopausal women with this achieving significance ($P = 0.04$) in the ductal components (Figure 1c,d). Examination of EGFR in normal tissue showed a consistently high expression of cell membrane staining in both ducts and lobules (Plate 1e,f). The staining was most evident towards the basement membrane, with luminal epithelial cells occasionally being recorded as negative. No obvious differences were observed in EGFR levels between pre and postmenopausal women and between ducts and lobules (not illustrated). Due to the high proportion of positive EGFR cells and their distribution in normal structures it was not possible to quantify EGFR measurements.

$Ki_{67}$ binding in all normal breast tissues examined was much lower than that recorded with the other antibodies with characteristically $<10\%$ cells immunostaining (Plate 1g,h, Figure 1e,f). Indeed in 25% of the samples no $Ki_{67}$ immunostaining was recorded. Highest levels of $Ki_{67}$ positivity were observed in the lobules removed from premenopausal women ($P = 0.02$).

### *Tamoxifen treated normal breast tissue*

Examination of the above parameters in 15 postmenopausal and two premenopausal tamoxifen treated women showed that the antioestrogen did not influence ER expression in the lobular component of normal breast tissue in comparison to postmenopausal controls (median values 30 and 20 respectively). It did, however, result in a significant increase in the percentage of ER positive ductal breast cells with more homogenous staining patterns being established (median values 30, and five respectively for tamoxifen treated and control postmenopausal patients, Figure 2b). Treatment with the antioestrogen did not influence the immunostaining patterns for PgR, $Ki_{67}$ (Figure 2c–f) and EGFR (not illustrated). The mean $Ki_{67}$ positivity in tissues from all tamoxifen treated patients remained below 5%. No obvious influence of the duration of tamoxifen treatment was apparent on the expression of ER, PgR and $Ki_{67}$ (Figure 3).

### Discussion

The antioestrogen tamoxifen has been suggested as a suitable prophylactic agent for the prevention of breast cancer in women who are deemed to be at high risk of developing the disease. This is in spite of very little information being available concerning the actions of the antioestrogen on the parenchymal structures of the normal breast. In our current study we have been able to establish using a number of immunohistochemical markers of hormone and growth factor receptors and a cell proliferation marker, that although tamoxifen may upregulate ER expression in ductal structures removed from postmenopausal treated patients, it shows no stimulatory activity on either PgR levels, a well known oestrogen regulated protein (Katzenellenbogen *et al.*, 1987; Welshons *et al.*, 1987) or the important parameter of cell proliferation (Figure 2). This is despite each of these end points showing a degree of hormonal regulation in non-treated patients, with their levels being modified by menopausal status (Figure 1).

Since $Ki_{67}$ immunostaining occurs at low frequency in the breast tissue of postmenopausal women ($<5\%$ cell staining) we envisaged that any stimulatory activity of tamoxifen on cell proliferation should have been readily detected. The absence of any increases in the proportion of $Ki_{67}$ positive cells in any of the samples removed from women after both short (2 days to 1 month) and long ($>2$ months) term tamoxifen treatment (Figure 3) suggests no adverse actions of the drug on normal breast tissue. Although our current study was largely performed on postmenopausal women, two samples removed from tamoxifen-treated premenopausal women also showed no evidence of elevated rates of cell proliferation. The ER and PgR values recorded in these specimens, however, were above the median values for the group in both ducts and lobules.

The data recorded above is in contrast to the oestrogen-like activity of tamoxifen on the human pituitary gland, uterus and liver of postmenopausal women, where the drug reduces elevated gonadotrophin levels (Furr & Jordan, 1984), increases the karyopyknotic index of the uterus (Ferrazzi *et al.*, 1977) and increases the serum concentrations of several oestrogen-regulated liver proteins (Sakai *et al.*, 1978; Boccardo *et al.*, 1981; Fex *et al.*, 1981). These results reflect the complex pharmacology of tamoxifen which is species, tissue and cell type specific (Furr & Jordan, 1984) and whose properties can range from a full oestrogen with no antagonistic properties towards oestradiol, to a full antagonist with no oestrogenicity. Indeed, we have previously ascribed the former property to tamoxifen with regards to its action on the rat mammary gland where it is a full agonist on ductal development and stimulates a large proliferative response in the terminal end buds, the main growth regions of the gland (Nicholson *et al.*, 1988). These studies were, however, carried out on pubertal animals during the active growth phase of mammary gland development. In mature cycling animals tamoxifen acts as an antioestrogen causing atrophy of lobular structures (Gotz *et al.*, 1984). In this light it may be significant that tamoxifen treatment of postmenopausal women resulted in some upregulation of the proportion of ductal epithelial cells expressing ER. Since, a similar phenomenon occurs in the transition between pre- and postmenopausal women, in both the normal (Figure 1) and

**Plate 1** Normal breast cells in ductal (**a,c,e,g**) and lobular (**b,d,f,h**) structures immunostained for ER (**a,b**), PgR (**c,d**), EGFR (**e,f**) and Ki67 (**g,h**).

**Figure 1** Influence of the menopause on immunostaining patterns in breast tissue. The data are presented as the percentage of normal breast cells in lobular structures (**a**,**c**,**e**) and ducts (**b**,**d**,**f**) that are ER (**a**,**b**), PgR (**b**,**c**) and Ki67 (**e**,**f**) positive and are subdivided according to the menopausal status of the patient. Mean values for the groups are shown by the dotted lines. The $P$ values calculated for differences between pre- and post-menopausal groups are **a**, $P = 0.02$, **b**, $P = 0.53$, **c**, $P = 0.06$, **d**, $P = 0.04$, **e**, $P = 0.02$, **f**, $P = 0.43$.

cancerous breast (Walker *et al.*, 1988), it is possible that the further increase observed in tamoxifen treated women may reflect a more efficient reduction in the availability of oestrogens to the tissue mediated by the antioestrogenic actions of the drug.

High levels of EGFR expression were evident in all normal breast tissue from both treated and untreated patients. Staining was, however, more frequently associated with the basal component, with luminal cells sometimes appearing EGFR negative. In view of the high level of EGFR positivity no correspondence was observed between its expression and immunostaining for ER, PgR and $Ki_{67}$. These data are in contrast to the inverse relationship between ER and EGFR expression observed in breast tumours (Sainsbury *et al.*, 1985), where EGFR immunostaining is associated with high grades of tumour malignancy (Lewis *et al.*, 1990). Moreover, in breast cancer specimens $Ki_{67}$ immunostaining also correlates with EGFR expression (McClelland & Nicholson, in preparation) and poor prognosis (Sainsbury *et al.*, 1987). These differences may result from the low availability of the ligands for the EGFR in normal tissues (Elder *et al.*, 1975; Kaselberg *et al.*, 1985; Poulsen *et al.*, 1986) and the presence of readily detectable amounts of TGF-α, the tumour homologue of EGF in the majority of breast cancers (Macias *et al.*, 1989).

In conclusion, the data presented do not show any adverse effects of tamoxifen on normal breast tissue. Most importantly treatment with the antioestrogen does not appear to stimulate cell proliferation even on long-term therapy. These data are therefore reassuring when considering the use of tamoxifen in ostensibly normal women who are deemed to be at high-risk of developing breast cancer.

**Figure 2** Influence of tamoxifen on immunostained patterns in breast tissue. The data are presented as the percentage of normal breast cells in lobular structures (**a**,**c**,**e**) and ducts (**b**,**d**,**f**) that are ER (**a**,**b**), PgR (**b**,**c**) and Ki67 (**e**,**f**) positive in tamoxifen treated (40 mg $d^{-1}$) and control patients. The majority of women receiving tamoxifen were postmenopausal (●) and are compared with control postmenopausal patients. The results obtained from two premenopausal women treated with the antioestrogen are illustrated by the symbol (▲). Mean values for the groups are shown by the dotted lines. The $P$ values calculated for differences between control and tamoxifen treated groups are **a**, $P = 0.23$, **b**, $P = 0.005$, **c**, $P = 0.80$, **d**, $P = 0.17$, **e**, $P = 0.28$ and **f**, $P = 0.47$.

**Figure 3** Influence of the duration of tamoxifen therapy on immunostaining patterns in breast tissue. The data are presented as the percentage of normal cells in ducts (●) and lobules (▲) that are ER, PgR and Ki67 positive in tamoxifen treated women as a function of the duration of therapy. The results obtained from premenopausal are shown by the □ symbol.

The authors wish to thank the Tenovus Organisation for their generous financial support.

### References

BOCCARDO, F., BRUZZI, P., RABAGOTTI, A., NICOLA, G. & ROSSO, R. (1981). Oestrogen liberation of tamoxifen on vaginal epithelium in breast cancer patients. *Rev. Endo. Rel. Cancer Suppl.*, **9**, 242.

BOUZUBAR, N., WALKER, K.J., GRIFFITHS, K. & 4 others (1989). Ki67 immunostaining in primary breast cancer: pathological and clinical associations. *Br. J. Cancer*, **59**, 943.

ELDER, J.B., WILLIAMS, G., LACEY, E. & GREGORY, H. (1978). Cellular localization of human urogastrone/epidermal growth factor. *Nature*, **271**, 466.

FENTIMAN, I.S. (1989). The endocrine prevention of breast cancer. *Br. J. Cancer*, **60**, 12.

FERRAZZI, E., CARTEI, G., MUTARAZZO, R. & FIORENTINO, M. (1977). Oestrogen-like effect of tamoxifen on vaginal epithelium. *BMJ*, **1**, 1351.

FEX, G., ADIELSSON, G. & MATTSON, W. (1981). Oestrogen-like effects of tamoxifen on the concentration of proteins in plasma. *Acta Endocrinol. (Copenh.)*, **97**, 109.

FURR, B.J.A. & JORDAN, V.C. (1984). The pharmacology and clinical uses of tamoxifen. *Pharmac. Ther.*, **25**, 127.

GERDES, J., LEMKE, H., BAISCH, H. & 4 others (1984). Cell cycle analysis of a cell proliferation associated human nuclear antigen defined by the monoclonal antibody Ki67. *Hematol. Oncol.*, **2**.

GERDES, J., LELLE, R.J., PICKCARTZ, H. & 4 others (1986). Growth fractions in breast cancers determined *in situ* with monoclonal antibody Ki67. *J. Clin. Pathol.*, **39**, 977.

GOTZE, S., NISHINO, Y. & NEUMANN, F. (1984). Anti-oestrogenic effects of tamoxifen on mammary gland and hypophysis in female rats. *Acta. Endocr. Copenh.*, **105**, 360.

KASELBERG, A.G., ORTH, D.N., GRAY, M.E. & STAHLMAN, M.T. (1985). Immunocytochemical localization of human epidermal growth factor/urogastrone in several human tissues. *J. Histochem. Cytochem.*, **33**, 315.

KATZENELLENBOGEN, B.S., KENDRA, K.L., NORMAN, M.J. & BERTHOIS, Y. (1987). Proliferation, hormonal responsiveness and estrogen receptor content of MCF-7 human breast cancer cells grown in the short-term absence of estrogens. *Cancer Res.*, **47**, 4355.

LEWIS, S., ELLIS, I.O., LOCKER, A. & 5 others (1990). Epidermal growth factor receptor expression in breast carcinoma. *J. Clin. Path.*, **43**, 385.

MACIAS, A., PEREZ, R., HAGERSTROM, T. & SKOOG, L. (1989). Transforming growth factor alpha in human mammary carcinomas and their metastases. *Anticancer Res.*, **9**, 177.

NATO REPORT. (1980). Controlled trial of tamoxifen as a single adjuvant agent in the management of early breast cancer. Analysis at 8 years. *Br. J. Cancer*, **57**, 608.

NICHOLSON, R.I., GOTTING, K.E., GEE, J. & WALKER, K.J. (1988). Actions of oestrogen and antioestrogens on rat mammary gland development: relevance to breast cancer prevention. *J. Steroid Biochem.*, **30**, 95.

PATTERSON, J.S., EDWARDS, D.G. & BATTERSBY, L.A. (1981). A review of the international clinical experience with tamoxifen. *Jap. J. Cancer Clin. Suppl. (Nov)*, 157–183.

PIKE, M.C., ROSS, R.K., LOBO, R.A. & 4 others (1989). LH-RH agonists and the prevention of breast and ovarian cancer. *Br. J. Cancer*, **60**, 142.

POULSEN, S.S., NEXO, E., SKOVOLSEN, P., HESS, J. & KIRKEGAARD, P. (1986). Immunohistochemical localization of epidermal growth factor in rat and man. *Histochemistry*, **85**, 389.

POWLES, T.J., HARDY, J.R., ASHLEY, S.E. & 4 others (1989). A pilot trial to evaluate the acute toxicity and feasibility of tamoxifen for prevention of breast cancer. *Br. J. Cancer*, **60**, 126.

REPORT FROM THE SCOTTISH BREAST CANCER TRIALS. (1987). Adjuvant tamoxifen in the management of operable breast cancer. The Scottish Trial. *Lancet*, **ii**, 172.

SAINSBURY, J.R.C., FARNDON, J.R., SHERBET, G.V. & HARRIS, A.L. (1985). Epidermal growth factor receptors and oestrogen receptors in human breast cancer. *Lancet*, **??**, 364.

SAINSBURY, J.R.C., NEEDHAM, G.K., MALCOLM, A., FARNDON, J.R. & HARRIS, A.L. (1987). Epidermal growth factor receptor status as a predictor of early recurrence of and death from breast cancer. *Lancet*, **i**, 1398.

SAKAI, F., CHEIX, F., CLAVEL, M. & 4 others (1978). Increases in steroid binding globulin induced by tamoxifen in patients with carcinoma of the breast. *J. Endocrinol.*, **76**, 219.

WALKER, K.J., BOUZUBAR, N., ROBERTSON, J. & 4 others (1988). Immunocytochemical localisation of estrogen receptor in human breast tissue. *Cancer Res.*, **48**, 6517.

WELSHONS, W.V. & JORDAN, V.C. (1987). Adaption of oestrogen-dependent MCF-7 cells to low oestrogen (phenol red free) culture. *Eur. J. Cancer Clin. Oncol.*, **23**, 1935.